# Supplementary material for: The role of spontaneous and evoked neuronal activity in protection from impending ischemic stroke during the hyperacute state
Source: Sci Rep. 2025 Jul 1;15:21705. doi: 10.1038/s41598-025-05620-x (PMC12217715; doi:10.1038/s41598-025-05620-x)
Supplement: Supplementary file 1 — Supplementary Material 1 [file 41598_2025_5620_MOESM1_ESM.docx]

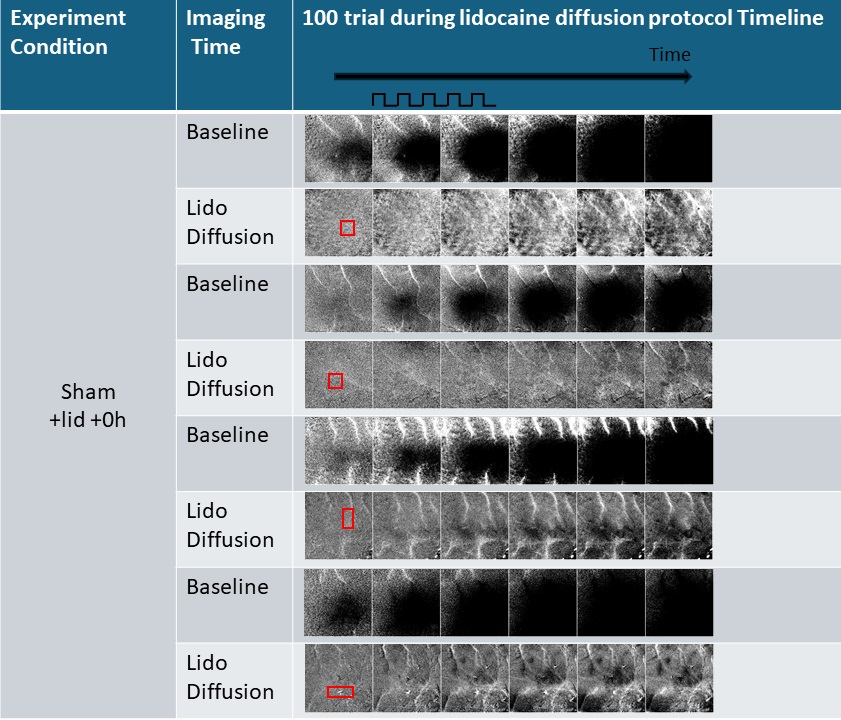


**Supplementary Figure 1.** *Four representative results of ISOI from group 2 (sham+lido+stim) using condensed whisker stimulation protocol. Red boxes in prestimulus frame indicate area of lidocaine diffusion. All images are 6mm by 6mm.*
